# Supplementary material for: A high-resolution mRNA expression time course of embryonic development in zebrafish
Source: eLife. 2017 Nov 16;6:e30860. doi: 10.7554/eLife.30860 (PMC5690287; doi:10.7554/eLife.30860)
Supplement: Supplementary file 6. [file elife-30860-supp6.zip › biolayout-clusters-files/Cluster173.html]

Cluster173


# Cluster173: Detail

### Go to ZFA detail

## GO

| GO ID | Description | Domain | Annotated | Expected | Observed | Adjusted p-value | Genes | Ensembl IDs |
| --- | --- | --- | --- | --- | --- | --- | --- | --- |
| GO:0000077 | DNA damage checkpoint | biological\_process | 16 | 0.01 | 2 | 0.0173 | fanci chek1 | ENSDARG00000026224 ENSDARG00000104765 |
| GO:0000086 | G2/M transition of mitotic cell cycle | biological\_process | 10 | 0.01 | 2 | 0.0063 | fanci chek1 | ENSDARG00000026224 ENSDARG00000104765 |
| GO:0008630 | intrinsic apoptotic signaling pathway in... | biological\_process | 15 | 0.01 | 2 | 0.0150 | atr chek1 | ENSDARG00000079625 ENSDARG00000104765 |
| GO:1901991 | negative regulation of mitotic cell cycl... | biological\_process | 15 | 0.01 | 2 | 0.0150 | fanci chek1 | ENSDARG00000026224 ENSDARG00000104765 |
| GO:0044774 | mitotic DNA integrity checkpoint | biological\_process | 10 | 0.01 | 2 | 0.0063 | fanci chek1 | ENSDARG00000026224 ENSDARG00000104765 |
